# Postoperative monitoring of ovariohysterectomy carried out with two types of surgical sutures in shelter cats and differences in selected blood parameters between shelter and pet cats

**DOI:** 10.1186/s12917-024-04177-7

**Published:** 2024-07-31

**Authors:** Jacek Cymbryłowicz, Paulina Jawor, Heliodor Wierzbicki, Piotr Trębacz, Tadeusz Stefaniak

**Affiliations:** 1 Gliwicka Przychodnia Weterynaryjna, Toszecka 19 Str, Gliwice, 44-100 Poland; 2 Department of Immunology, Pathophysiology and Veterinary Preventive Medicine, Wrocław University of Environmental and Life Sciences, C.K. Norwida 31 Str, Wrocław, 50-375 Poland; 3 Department of Genetics, Wrocław University of Environmental and Life Sciences, Kożuchowska 7, Wrocław, 51-631 Poland; 4 Department of Surgery and Anaesthesiology of Small Animals, Institute of Veterinary Medicine, Warsaw University of Life Sciences, Nowoursynowska 159 C, Warsaw, 02-776 Poland

**Keywords:** Acute phase response, Ovariohysterectomy, Absorbable, Non-absorbable sutures

## Abstract

**Background:**

Reduction of inflammation and early detection of complications after surgical procedures are important objectives for proper veterinary practice. This study aimed to evaluate the differences between shelter and pet female cats in selected acute-phase parameters scheduled to ovariohysterectomy. Postoperative monitoring after ovariohysterectomy with the same laboratory parameters was performed in shelter cats, in which two different types of surgical sutures were used for the entire procedure. The experimental group comprised 40 female cats from animal shelters (‘shelter cats,’ *n* = 40). These cats were divided into two subgroups: group A (*n* = 20) operated on with absorbable sutures and group NA (*n* = 20) operated on with non-absorbable sutures. In addition, the same parameters were evaluated in pet female cats (*n* = 19). Blood was collected from shelter cats immediately before surgery (term 0), at 24 and 72 h (terms 1 and 3, respectively), and at 7 and 14 days (terms 7 and 14, respectively) after ovariohysterectomy. Blood samples from the pet cat group were collected only once.

**Result:**

The mean haptoglobin concentration before ovariohysterectomy in pet cats was significantly lower than that in shelter cats. Fibrinogen concentration was significantly lower in pet cats than in cats from group A. Serum albumin, beta-1, beta-2, and gamma-globulin concentrations were significantly higher in the shelter cats than in the pet cats. Subcutaneous tissue thickening at the site of the postoperative wound was observed in five patients cats (25%) in group A, and two (10%) cats in the NA group.

**Conclusion:**

These results indicate that ovariohysterectomy leads to local and general inflammatory responses. The majority of cats from animal shelters suffered from subclinical inflammation.

## Background

In female domestic cats, ovariohysterectomy (OH) is one of the most commonly performed surgical procedures in veterinary practice. This procedure is considered a method of contraception for population reduction and for the prevention of diseases, such as mammary tumors [1]. The most commonly mentioned complications after OH are delayed healing of the surgical wound, abscesses or infections related to the use of surgical sutures, and self-injury to the surgical wound [2]. The aim of choosing the correct surgical suture is to ensure that the wound heals properly with as little tissue trauma as possible [3]. Inappropriate sutures can promote the development of wound infections and impair healing [4]. Therefore, selection of the right material is crucial for an uncomplicated healing process. Polyglycolic acid (PGA) (Dexon, Dexon II) is a braided synthetic absorbable suture made from a synthetic homopolymer of glycolic acid [5]. It hydrolyzes during biodegradation, and its degradation products are not good media for bacterial growth [5, 6]. Despite this, the polyfilamentous structure of Dexon promotes easier contamination than monofilament materials [4]. Nylon (polyamide) induces little tissue reaction, even in the tendon tissues [5].

Bacterial infections, tissue damage due to trauma or surgery initiate a number of defense and adaptive processes to maintain homeostasis. These mechanisms have been termed acute phase response (APR) and are manifested by changes in the plasma concentrations of acute phase proteins (APPs) [7]. The major APPs in felines are serum amyloid A (SAA) and alpha 1-acid glycoprotein (AGP), both of which increase a few hours after the inflammatory stimulus and remain elevated for as long as the inflammation persists [8]. In cats, there is an increase in haptoglobin (Hp) concentrations during natural and experimentally induced diseases [9]. Hp levels are positively correlated with serum fibrinogen (Fb) levels and leukocyte concentrations [10]. There are no data on the possible role of albumin as a negative acute-phase protein in cats [8], but in many feline inflammatory conditions, it is reported to be decreased [11, 12]. Fever is a common finding in cats with inflammation, with leukocytosis caused by the release of cells from the marginal pool, followed by activation of myelopoiesis [8]. Only a few studies have monitored queens after OH by using APP have been done. The number of animals in these studies was either scarce [13, 14] or only determined before surgery, but not after [15].

This study aimed to evaluate the dynamics of selected acute-phase proteins when two types of surgical sutures were used and the usefulness of acute-phase proteins in the early diagnosis of postoperative complications in female cats. In addition, the concentrations of APPs before surgery in pet and shelter cats were compared.

## Results

Among the cats undergoing surgery, pregnancy was noted intraoperatively in three individuals from group A (15%) and six from group NA (30%). During daily observations, 4–8 days (mean 6.4 ± 1.3) after surgery, complications were noted in five patients cats (25%) in group A, and two (10%) cats in the NA group. Subcutaneous tissue thickening appeared at the site of postoperative wounds in these animals. During the clinical examination, these lesions were nonpainful and immovable. The skin wound healed properly, with the exception of one female cat (from group A), which showed seropurulent discharge from the wound. By the end of the observation period, the thickenings decreased in size, and their consistency became more firm than at the beginning. The lesions did not cause any other clinical signs compared with the animals in which the postoperative period was uncomplicated. Ratio of odds for tissue thickening in A group was 3 (95% CI 0.5–17.7, *P* > 0.05).

The results of the determination of acute phase protein and total serum protein fractions are shown in Table 1. After OH on day 3, there was an increase in SAA in both groups; however, this difference was not statistically significant. The mean Hp concentration in pet cat group was lower than that in both groups of shelter cats (Table 1, *P*≤ 0.05). Immediately before surgery, the highest Hp concentration was observed in the A cat group, which was significantly higher than that observed in the NA group. With the exception of day 1 post-treatment, the Hp concentration was significantly different (*P*≤ 0.05) at all subsequent times (i.e., days 0, 3, 7, and 14) between the experimental groups (NA and A). A high Hp concentration was observed on day 3 after treatment in both groups, which was significantly different from the Hp concentration determined on day 14 in both groups. The Fb concentration immediately before surgery was lower in the pet cats than in the shelter cats in which surgery was performed with absorbable sutures (Table 1, *P*≤ 0.05). Group A had significantly higher fibrinogen concentrations than group NA throughout the follow-up period. In each group, there was no significant increase in fibrinogen concentration owing to OH. Higher albumin concentrations were observed in pet cats than in the experimental groups (*P*≤ 0.05, Table 1). Despite numerically higher concentrations of the alpha-globulin fraction, the experimental groups were not significantly different. Beta-1 and beta2-globulin concentrations were significantly higher in shelter cats than in pet cats. Beta-1-globulins were significantly higher in the NA group than in the A group throughout the observation period. In the pet cat group, the mean gamma-globulin concentration was more than 2-fold lower than that in the experimental groups. This difference was statistically significant (*P*≤ 0.05) (Table 1). In both shelter groups, OH treatment did not lead to any significant changes in albumin, alpha-, beta-, or gamma-globulin concentrations.

**Table 1:**
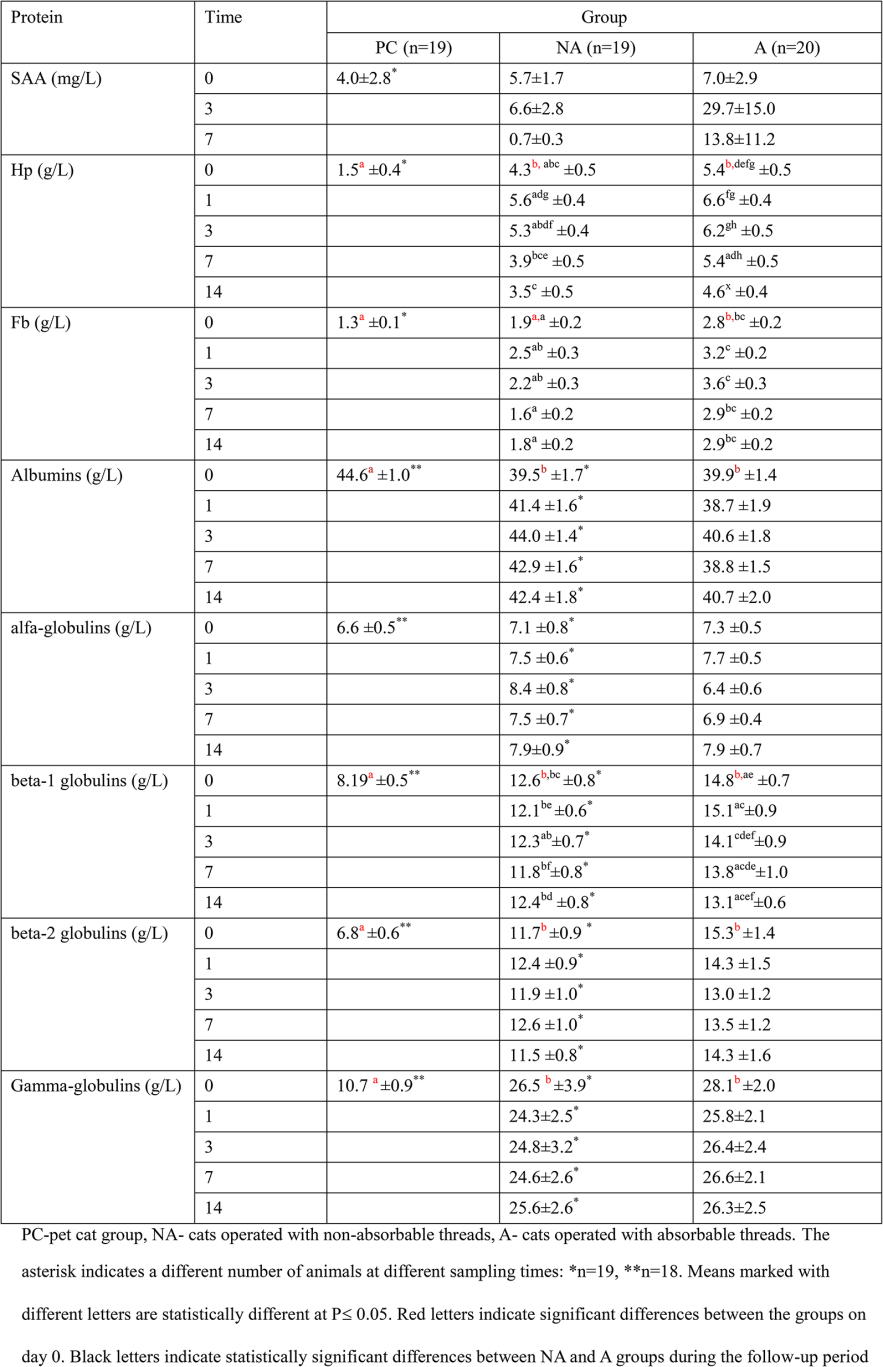
Concentration of acute-phase proteins and serum protein fractions in examined groups during study

None of the parameters listed in Table 2 showed a significant increase after the OH treatment. The number of erythrocytes in the pet group was not significantly different from that in the experimental groups and there were no differences between the experimental groups at the same sampling time. The number of leukocytes was lower in the pet cat group than that in the experimental groups (*P*≤ 0.05; Table 2).

**Table 2:**
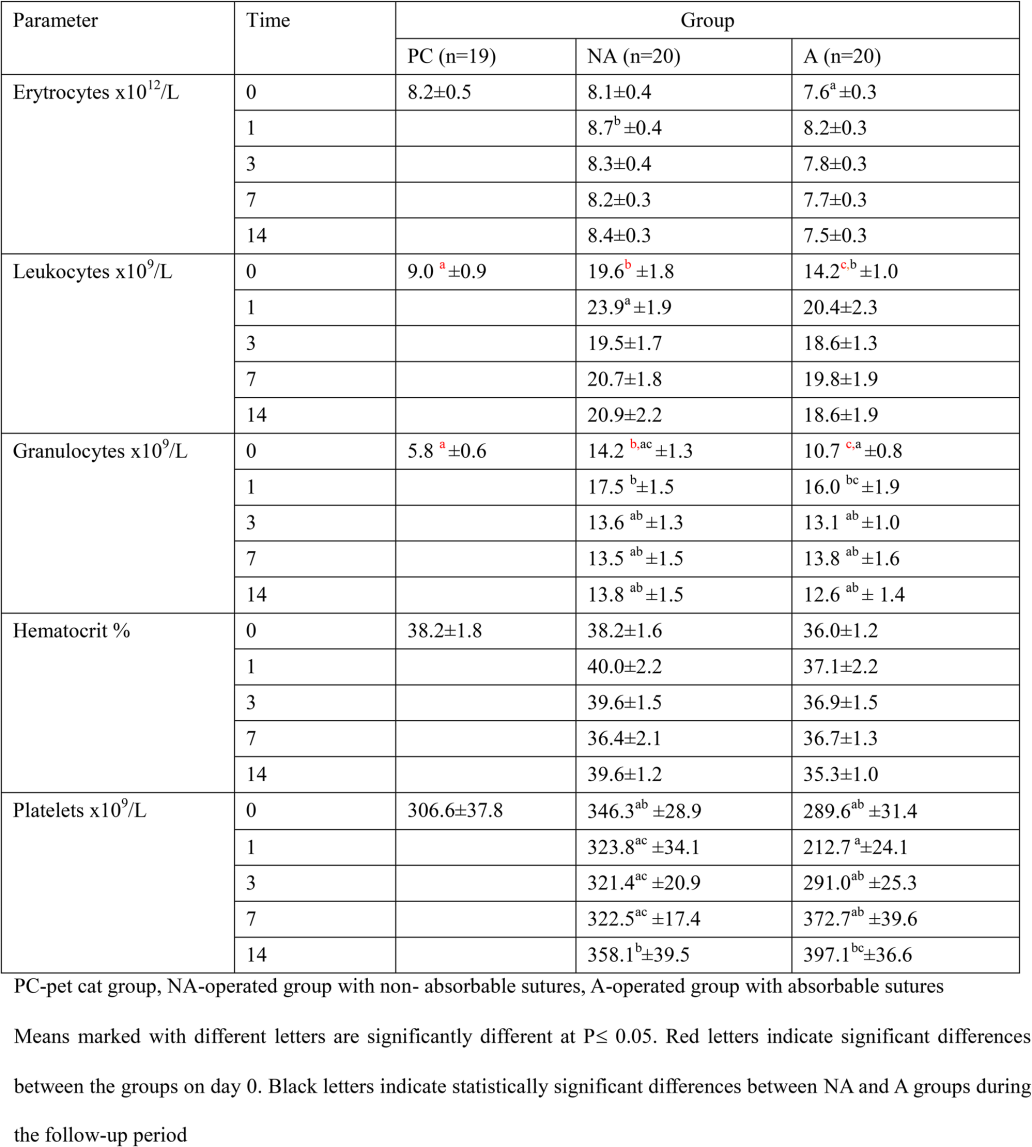
Concentration of selected blood parameters in examined groups during study

The largest fraction of leukocytes were granulocytes, which were lower in pet cats than in shelter cats (*P*≤ 0.05; Table 2). In addition, cats operated with non-resorbable suture threads had a larger count of granulocytes than other shelter cats (*p*≤ 0.05; Table 2). After surgery, there was an initial significant increase in leukocyte count on day 1, and at the following sampling times, the values were not significantly different. The hematocrit values did not differ between the groups during the study. Platelet counts did not differ between the groups; only a significantly higher platelet count was found in the NA group on day 14 than on days 1, 3, and 7 after treatment (Table 2, *P*≤0.05). In Group A, the platelet count was higher on day 14 than on day 1 after surgery (Table 2, *P*≤ 0.05).

## Discussion

In this study, the usefulness of various parameters in assessing inflammation during the postoperative period was evaluated. The concentration of SAA in cats before OH surgery was low. In cats in which absorbable sutures were used, the SAA concentration increased on the third day after surgery and then decreased on the seventh day, but these changes were not statistically significant. Sasaki et al. [13] also demonstrated increases in SAA within 3–6 h after ovariohysterectomy in cats, with a peak at 24 h; however, it was evaluated only in three cats. Due to the lack of SAA determination on postoperative day 1, it is not known whether SAA concentrations were highest on the first postoperative day in the our study. However, in the days following surgery, SAA showed similar dynamics to those reported by Kajikawa [10], where SAA concentrations slowly decreased to baseline values from days 4 to 13. In a study by Shida et al. [14], SAA reached maximum values at a similar time i.e. 1–2 days after OH surgery, but returned to normal quicker (within 5 days); however, the study group included only five cats. In this study, on the third day after surgery, the mean SAA concentration was almost four times higher in cats treated with absorbable sutures than in those treated with non- absorbable sutures. SAA is a rapidly reacting protein [10, 16]; therefore, surgical trauma caused an immediate increase in SAA production, which was more intense in group A. The lack of statistical difference indicates that more injury occurred only in some individuals in group A. Multifilament threads, owing to their properties, can increase the risk of infection [4] and can also mechanically damage the tissue through which they pass, creating a so-called saw effect [17]. They elicit a stronger local reaction in tissues than monofilament threads, which may explain the more intense inflammatory reaction in some animals. It also cannot be ruled out that there was a large variation in the concentration of this protein between individuals on the third day after surgery, which may have been because the production of acute-phase proteins in animals varies between individuals [18] and that the concentration of acute-phase proteins largely depends on the extent and nature of the inflammatory reaction [19]. On day 7 after surgery, numerically higher SAA concentrations were observed in Group A, but this could be the consequence of high concentration of SAA in some cats on day 3.

Haptoglobin is a positive acute-phase protein that is frequently determined in cats [16]. Hp concentrations were significantly higher in shelter cats than in pet cats, which may be because of their environment of origin. Shelter cats are also exposed to a wide range of viral and bacterial pathogens. Haptoglobin is not pathogen specific acute-phase protein [20]. This increase can also occur in aseptic inflammation after trauma, in cases of tissue necrosis, gastrointestinal tract inflammation, or in certain metabolic disorders and stress [21–23]. Therefore, despite the absence of obvious clinical signs (apart from reddening of the mucous membranes in some cats), animals may be in a state of chronic infection and thus have higher serum Hp concentrations [24]. On the first postoperative day, a numerical increase in Hp concentration was observed (not statistically significant) in the A and NA groups, which could be explained by an increase in the concentration of this protein in response to surgical trauma associated with the OH procedure performed. On this day, the determined Hp concentrations were the highest during the entire observation period. Thus, the dynamics of Hp concentrations differed slightly from the results of Kajikawa [10], who studied Hp concentrations after urinary tract surgery. The cited author recorded a significant increase in Hp levels on the first postoperative day; the highest concentration was found only on the second postoperative day. The differences in the dynamics of Hp concentration after surgery in the assessment of the postoperative period in our study compared to the results of Kajikawa [10] are most likely due to the fact that, in the present study, blood was not collected on the second day, but only on the third postoperative day, so it is not excluded that Hp concentration may have been even higher on the second day in the present study. Subsequently, on the following days, the dynamics were similar to those reported by Kajikawa [10], and the Hp concentration decreased gradually until day 14 after surgery. In cats with thickened subcutaneous tissue in the area of the incision site (cats classified as suffering from a complicated postoperative period), Hp concentrations were, on average, higher than those in cats without complications from day 1 after surgery until the end of the follow-up period (data not shown). At the last follow-up date (day 14 after surgery), the mean Hp concentration in group A was significantly lower than that before surgery, which could be explained by the effect of the antibiotic and NSAID drugs, as well as by keeping the cats probably under conditions with a higher standard of hygiene than in the shelter. Cats classified in group A initially had significantly higher Hp concentrations than cats in which NA threads were used. The pharmacological therapy undertaken probably had the effect of reducing chronic infections accompanying the shelter animals prior to surgery and had a similar effect to that of SAA, although still in the last sampling the Hp concentration in the A group was significantly higher than that in the NA group.

In our study, the mean Fb concentration varied slightly in the experimental groups after treatment and did not exceed the reference limit of 2–4 g/L [25]. It can be seen that in pet cats, the Fb concentration on day 0 was significantly lower than that in cats from the shelter (group A), which, as with other proteins, is probably due to the different housing conditions and antigenic pressure of the environment. Also in cats treated because of urinary obstruction initial fibrinogen median concentration exceeded about 50% that of control group (2 g/L) and decreased from 3,11 to 2,75 g/L during 48 h after intervention [12]. The low dynamics of change in this protein during the postoperative period indicate little usefulness in patient monitoring.

In this study, the analgesic protocol consisted of a single dose of tolfenamic acid. In cases where rescue analgesia is needed after ovariohysterectomy, the response of acute-phase proteins should not be disturbed, since nonsteroidal anti-inflammatory drugs reduce prostaglandin release without interfering with the inflammatory pathway [26].

In cats, the role of albumin as a negative factor has not been proven [11]. This was shown in study Kann et al. [16], where albumin concentrations did not appear to differ between cats of varying health status. In our study, low variability was observed in all cats studied after OH, and the mean albumin concentration was at the upper end of the range of values obtained by Kann et al. [16] in healthy cats. It cannot be ruled out that the lack of a decrease in albumin concentration during the postoperative period was due to the change in diet of the cats, which were transferred from shelter conditions to individual cages for 2 weeks and were fed high-quality food at will for most of their stay. The improved protein content of the feed may have had a beneficial effect on albumin synthesis in the liver and offset any inhibition of this process associated with the acute phase reaction.

The concentrations of the studied protein fractions (albumin and alpha-, beta-, and gamma-globulin) did not change significantly within the group after treatment. The differences found, that is, significantly lower concentrations of albumin and significantly higher concentrations of both beta and gamma fractions in shelter cats than in pet cats, were due to the influence of the environment from which the animals originated. In a study by Kristensen and Barsanti [27], cats that lived in a research colony environment were found to have significantly increased levels of alpha- and gamma-globulins and decreased levels of albumins compared with cats kept as house pets. Similarly, in cats with infectious peritonitis (FIP), alpha − 2, beta-, and gamma-globulin levels were significantly higher and albumin levels were lower than those in healthy cats [25]. The significantly higher beta-1 and beta-2 fraction scores of shelter cats may have been influenced by elevated concentrations of proteins that migrate in this fraction, such as apolipoprotein, complement C3, fibronectin, lactoferrin, and IgM and IgG immunoglobulins [28]. The latter may also migrate to the gamma fraction [24], leading to higher concentrations of this fraction. However, hypergammaglobulinemia is not necessarily related to antibody titers, suggesting the presence of other proteins with gamma motility (e.g., complement fractions) [29]. High concentrations of gamma- globulin fractions in cats were also found during *Trypanosoma evansi* invasion [30]. Owing to the lack of differences between the groups after surgery, it appears that the determination of individual fractions is not useful in monitoring the postoperative period in cats, although determination before surgery to assess the stimulation of the cat’s immune system may be useful.

Another indicator of the acute phase response in cats is leukocytosis [31]. Dabrowski and Wawron [32] found a significant increase in mean leukocyte counts on the first day after uterine and ovarian amputation in healthy bitches compared with their baseline values, which is a consequence of the development of a local inflammatory reaction resulting from tissue disruption due to surgery, along with the activation of the systemic acute phase response [33]. In our study, cats also showed an increase in leukocyte counts on the first day after OH, similar to the findings of Dabrowski and Wawron [32]. The increase in leukocytes was influenced by a statistically significant increase in the number of granulocytes, the most abundant subpopulation of blood leukocytes, which respond most rapidly to inflammation. In subsequent blood sampling, leukocyte and granulocyte counts decreased, and the differences between the groups at a given follow-up date were not statistically significant. Sergeeff et al. [34] showed that, in cats, an increase in granulocytes rather than leukocytes is the most characteristic of an inflammatory response. No differences in leukocyte or granulocyte counts were found based on the type of strand used for OH. Erythropenia is also found in the blood during the acute phase of chronic conditions [35]. Despite elevated gamma-globulin and leukocyte concentrations in shelter cats compared to those in pet cats, erythrocyte counts, hemoglobin concentration, and hematocrit values did not differ between the groups. This indicated that the red cell system was not affected by the ongoing inflammatory processes. The number of erythrocytes was not significantly different between the groups on successive control dates. The highest hematocrit value was found on the first day after surgery, which is understandable given the limited water intake before and after surgery. Notably, erythrocyte count, Ht value, and platelet count were not significantly affected by surgery. Therefore, the usefulness of these parameters for monitoring the postoperative period appears to be limited. Similarly, in a study by Fazio et al. [36], haematological variables one hour after surgery were not significantly affected by ovariohysterectomy in clinically healthy cats.

In our study, 10% of cats in the NA group and 25% in the A group had such a complications, while the total percentage of observed complications among all operated cats was 17.5%. This percentage is similar to the results of a retrospective study by Pollari et al. [37], who studied the postoperative period in cats after OH but used only absorbable sutures. However, it should be noted that this study was conducted with not only absorbable but also non- absorbable sutures, after which the complication rate was lower. In an experiment on 99 cats, Freeman et al. [38] found, 5–7 days after OH, inflammation and connective tissue proliferation can cause swelling at the level of the incision line. The swelling may contain 0.5-2.0 ml of serous-bloody fluid. In the cited study, wounds after surgery were studied using different absorbable surgical sutures. No correlation was found between the occurrence of complications and the type of suture; however, it was noted that not placing sutures in the subcutaneous tissue generated fewer complications. A similar conclusion was drawn by Muir et al. [39], who noted a clear relationship between the location of sutures in the subcutaneous tissue and the observed swelling of the subcutaneous tissue. In this study, one of the possible causes of subcutaneous tissue thickening may be the use of sutures in the subcutaneous tissue. Another reason could be the type of suture thread that was used. Absorbable sutures, compared with non-absorbable sutures in cases of intradermal skin closure in cats, induce longer-lasting and more prevalent incisional swelling [40]. This may have resulted in a more frequent finding of subcutaneous tissue thickening in Group A.

In summary, the results of this study showed that OH as a surgical procedure leads to the development of local and general inflammation. This was manifested by an increase in SAA, Hp, and Fb concentrations and granulocytosis. Some parameters - albumin concentration, gamma-globulin, erythrocytes, Hb, and Ht values–associated with OH showed minor changes.

## Conclusions

Based on the Hp, beta-fractions, and gamma-globulin levels, it can be concluded that most cats from animal shelters had subclinical inflammation at the time of OH surgery. After ovariohysterectomy, cats operated on with polyfilament absorbable sutures slightly more often develop thickening in the area of the surgical wound than those operated on with monofilament sutures; therefore, cats whose postoperative care may be difficult should be operated on with monofilament sutures.

## Materials and methods

### Animals

All the cats were European shorthair cats. The experimental group consisted of cats from four animal shelters in Upper Silesia, Poland (‘shelter cats,’ *n* = 40). In shelters, cats were selected for surgery if they were considered healthy by their caregivers. Two days before the planned surgery, they were subjected by their keepers to prophylactic flea control with fipronil (Fiprex cat; Vet Agro, Poland) and deworming with praziquantel and pyrantel embonate (Pratel; Animal Health, 1 tablet /10 kg body weight). Upon arrival at the clinic, the animals underwent a clinical examination, during which the nutritional status, skin condition, coat quality, appearance of the oral mucosa, conjunctival appearance, discharge from the conjunctival sacs, corneal condition, and internal body temperature were recorded. The chest was auscultated and the abdominal cavity was palpated. Otoscopic examination of the external ear canal was performed. All animals were clinically examined, and none of the cats showed signs of disease, with the exception of conjunctival congestion in some individuals. Only cats in which pregnancy was not detected during palpation were eligible for inclusion in the study. These cats were divided into two subgroups: 2a) group A (*n* = 20) operated on with absorbable sutures and 2b) group NA (*n* = 20) operated on with non-absorbable sutures. Admission to group A or NA was based on the order in which the animals arrived at the clinic. Treatments began by performing all OH in sequence with absorbable sutures, followed by the group treated with non-absorbable sutures.

The pet cat group, from which blood was collected once for comparison purposes, consisted of cats from private owners (so-called “pet cats”, *n* = 19), whose health status was checked before the planned (1–3 days) ovariohysterectomy. However, blood was not drawn several times in these cats as part of monitoring the healing process. This was due to the fact that the owners took these cats home after surgery, and it was not possible to bring them to the clinic at planned dates for monitoring, whereas cats from shelters stayed at the clinic for 14 days.

All cats were intact females aged 1–3 years (mean, 2 years), with body weight ranging from 2.4 to 4.5 kg (mean 3.45 kg). Owing to the lack of precise information on the age of animals referred from shelters, it was assessed based on dental status, body shape, and degree of mammary gland development.

### Ovariohysterectomy procedure

Ovariohysterectomy was performed in 2011. Prior to surgery, the animals were subjected to a 12-hour fasting. Access to water was not restricted. Each cat received the same set of drugs at doses proportional to the body weight. General anesthesia was induced and maintained with Sedazin (xylazine hydrochloride; Biowet Pulawy) 0.5-1 mg/kg b.w. intramuscularly, followed by Bioketan (ketamine hydrochloride; Vetoquinol Biowet sp. z o.o.) 5–10 mg/kg b.w. intramuscularly. After surgery, they received procaine benzylpenicillin and benzathine benzylpenicillin (Penicillin LA, Norbrook Lab. Ltd. Station Works) 0.1 ml/kg b.w. subcutaneously, and tolfenamic acid (Tolfedine 4%, Vetoquinol Biowet sp. z o.o.) 4 mg/kg b.w. subcutaneously. Dexon II 3 − 0, bicolor (braided, polyglycolic acid coated with Policaprolate^®^, prod. Syneture, catalogue no. 9801-41) in group A or Amifil M 3 − 0 (monofilament, polyamide, prod. Sinpo code 2MG 302) was used for the NA group. After preparation of the operating field (shaving the skin in the area from the processus xiphoideus to the pubic brim, the skin was washed with 70% ethanol solution and disinfected with 2% iodine tincture), the animals were placed on their backs. A celiotomy was performed on the ventral midline, encompassing the cranial third of the distance between the umbiculus and the pubis. The uterus was located by using an ovariohysterectomy hook. After ligation and transection of both the ovarian pedicles, the mesometrium was transected. Uterine body ligation and transection were performed caudally to the uterine bifurcation. The residual endometrium was cauterized using bipolar forceps. The linea alba, subcutis, and skin were closed in layers. Two layers of suture were placed. The linea alba and subcutis were sutured together and the skin was sutured separately using a single horizontal mattress suture. Sutures were removed after 10 days.

Cats from subgroups A and NA were kept in the hospital of the clinic under 24-hour observation throughout the observation period (until day 14 after surgery). Each individual was housed in a separate stainless steel cage and received the same care. The animals were fed dry food (Purina Pro Plan, Housecat, Home Catschicken, PA, USA). The cages were washed daily, and the litter trays were cleaned.

Each cat in the experimental group was clinically examined before surgery (term 0), at 24 and 72 h (terms 1 and 3), and 7 and 14 days (terms 7 and 14) after surgery. The condition of the conjunctiva and cornea, and the presence of any discharge from the conjunctival sacs were assessed. The oral mucous membranes, number and quality of respiration, and behaviour of the cats in the cage were assessed. Venous blood samples were collected from each cat on the same day. Blood was collected from the saphenous, femoral, or cephalic veins. Approximately 0.5 ml of blood was collected at one time for anticoagulant (K2 EDTA) and approximately 3 ml into a tube without anticoagulant to obtain serum. Owing to the aggressive behaviour and high stress levels of some individuals, internal temperature testing was abandoned. Throughout their stay at the clinic, the cats were observed daily by the keepers, and attention was paid to behaviour, food and water intake, as well as fecal and urinary output. The surgical wound was inspected and palpated to detect complications. Observations were noted in patient records.

### Laboratory analysis

Hematological blood analysis (erythrocytes, leukocytes, granulocytes, hematocrit, and platelet counts) was performed using the ABC Vet (Horiba) and fibrinogen levels (according to Millar et al. [41]) were determined immediately after blood collection. Serum samples were obtained by centrifugation at 2000 × g, separated into three Eppendorff tubes, and stored at -20^o^C for further analysis. The following were determined in the serum: haptoglobin concentration (guaiacol method [41]), serum amyloid A (ELISA from Tridelta Ltd.), and total serum protein and its fractions (paper electrophoresis). The SAA assay was performed on samples collected at time points 0, 3, and 7, whereas the other assays were performed at all time points. The intra-assay CV values for Hp and SAA were 3.3% and 9.7%, respectively. The inter-assay CV for Hp and SAA were 5.8% and 5.1%, respectively.

### Statistical analysis

The collected data were statistically analyzed using the non-orthogonal ANOVA with the least-squares method (lsmeans). The Shapiro-Wilk test was used to test whether the data were normally distributed. The significance of the differences between the means (*p*≤ 0.05) was tested using the Bonferroni multiple comparison test. The Bonferroni correction was applied to adjust probability (p) values because of the increased risk of a type I error when making multiple comparisons. All statistical analyses were performed using the R package. For clarity of the presented results and the possibility of comparing them with literature data and reference ranges, they are presented in tables as arithmetic means, together with the standard error (SE). Significance of differences is presented in uppercase letters at *P*≤ 0.05.

## Data Availability

All data generated or analyzed during this study are available upon request from the corresponding authors.
